# A historical review of ephaptic field research: from early foundations through contemporary renaissance

**DOI:** 10.3389/fnhum.2026.1747899

**Published:** 2026-04-13

**Authors:** Tam Hunt, M. Bruce MacIver

**Affiliations:** 1 University of California, Santa Barbara, Santa Barbara, CA, United States; 2 Stanford University, Stanford, CA, United States

**Keywords:** brain networks, consciousness, electromagnetic fields, ephaptic coupling, neural communication

## Abstract

Ephaptic field research has undergone a remarkable evolution spanning over nine decades, from pioneering observations in the 1930s through a period of severe scientific skepticism in the mid-20th century to a contemporary renaissance driven by advanced computational modeling, measurement techniques, and new consciousness theories. This review traces the complete chronological development of ephaptic coupling research, examining early foundational work by Adrian, Katz and Schmitt, and Arvanitaki, the influential skepticism of Lashley’s study that marginalized the field for decades, and the recent resurgence beginning in the 2000s that has led to recognition of ephaptic interactions as providing fast and direct communication throughout the brain. Contemporary research has established that weak electric fields (0.1–5 V/m) can produce measurable physiological effects and that ephaptic coupling contributes significantly to brain network complexity, memory formation, and potentially consciousness itself. Ephaptic communication, together with some form of electromagnetic field (EMF) theory of consciousness, provides a ready solution to the critical ‘binding problem’ that has perplexed philosophers and neuroscientists for at least the last century. This historical perspective demonstrates how scientific paradigms can shift dramatically as methodological advances allow for more sophisticated investigation of previously dismissed phenomena.

## 1 Introduction

This mechanism provides rapid, bidirectional communication through quasistatic electromagnetic fields. Importantly, action potential propagation itself is a longitudinal electromagnetic coupling phenomenon along the neuronal membrane, while ephaptic interactions represent transverse electromagnetic coupling through surrounding tissue. Both originate from the same unified electromagnetic field system generated by neuronal activity.

This review provides a comprehensive chronological examination of ephaptic field research, documenting the complete arc of scientific development in this field. Throughout, we employ precise electromagnetic terminology: “quasistatic” (near-field) electromagnetic fields that propagate at light speed through neural tissue, distinguished from “radiative” (far-field) electromagnetic effects. We clarify that both action potential and ephaptic coupling phenomena arise from a unified electromagnetic field system with longitudinal (along membrane) and transverse (through tissue) propagation components.

Table 1 summarizes the phases of research in this field.

**Table 1:** Historical phases of ephaptic field research.

Phase	Time period	Key researchers	Major findings	Significance
Early foundations	1930s–1940s	Adrian (1936), Katz and Schmitt (1940), Arvanitaki (1942)	First systematic evidence for electrical interactions between adjacent nerve fibers; coined term “ephaptic interactions”; demonstrated action currents could evoke responses with 2–5 ms delays; established “preephaptic” and “postephaptic” terminology	Established conceptual and experimental foundations for field-based neural communication as distinct from synaptic transmission
Mid-century skepticism	1950s–1970s	Lashley et al. (1951), Eccles and Jaeger (1958)	Metallic conductors in visual cortex showed no functional disruption; Lashley concluded electric fields “not an important factor in cerebral integration”; limited evidence for ephaptic effects at synaptic cleft level; field marginalized for decades	Effectively suppressed ephaptic field research; dominant synaptic paradigm prevailed; skepticism became entrenched in neuroscience community
Early revival	Mid-1980s	Richardson et al. (1984)	First clear demonstrations in mammalian cortical circuitry (hippocampal CA1); extracellular fields could ephaptically discharge neurons; field effects contributed to paired-pulse and frequency potentiation; used rigorous *in vitro* slice methodology	Provided early evidence that ephaptic effects were functionally relevant in cortex, though findings remained peripheral to mainstream neuroscience
Modern renaissance	2000s–2010s	Frohlich and McCormick (2010), Anastassiou et al. (2011), Buzsáki et al. (2012)	Exogenous fields as weak as 0.5 V/m could modulate network activity; endogenous fields could coordinate individual neuron spiking; fields of 0.6 mV extracellular and 6 mV/mm^−1^ comparable to natural conditions; strong entrainment for slow (<8 Hz) field fluctuations	Advanced computational tools and sophisticated recording enabled rigorous demonstration that ephaptic coupling was physiologically relevant under natural conditions
Breakthrough period	2019–2020	Chiang et al. (2019), Ruffini et al. (2020), Herweg et al. (2020)	Slow waves could propagate across 400 μm tissue gaps (impossible via synapses); definitive proof ephaptic coupling supports information transmission; finite element modeling of mesoscopic coupling in human brain; theta oscillations linked to memory formation via field effects	Provided unambiguous evidence that ephaptic coupling could transmit meaningful information; computational advances enabled realistic modeling at scale
Contemporary validation	2022–2024	Lee et al. (2024), Pinotsis and Miller (2023), Cunha et al. (2024), Hunt and Jones (2023)	Cell-class-specific electric field entrainment demonstrated; effects confirmed in human neurons and multiple brain regions; ephaptic coupling described as “alternative, energy-efficient, non-synaptic paradigm”; field effects contribute to brain complexity and memory network formation	Established ephaptic effects as fundamental mechanisms of neural coordination; shifted from skepticism to recognition as core computational principle

This table condenses over nine decades of research into distinct phases, though boundaries between phases are somewhat fluid. The progression demonstrates how technological advances, theoretical frameworks, and accumulating empirical evidence transformed ephaptic coupling from dismissed curiosity to recognized mechanism of neural computation.

This historical arc reveals several fundamental insights about scientific progress and paradigm shifts in neuroscience. First, the dismissal of ephaptic field theories for seven decades was not driven by failed experiments but by misinterpretation of experimental results—specifically, Lashley’s assumption about field geometry proved as important as the experimental data themselves. Second, the contemporary renaissance demonstrates that paradigm shifts often require not just new data but new tools for interpreting existing data: the same phenomena Lashley observed can now be understood through geometric and computational frameworks unavailable in 1951. Third, understanding consciousness may require a fundamental reconceptualization of how neural computation occurs—moving beyond spike-centric explanations to recognize that various scales of electromagnetic fields constitute the primary computational substrate through which neurons coordinate their activity at multiple scales.

## 2 Early foundations (1930s–1940s)

### 2.1 Pioneering observations

The idea that neurons could communicate via electric fields was proposed early in the 1900s, by a pioneer in neurophysiology, E. D. Adrian (1919). Empirical evidence for ephaptic communication trace to Adrian’s pioneering neurophysiological work in the 1930s (Adrian and Matthews, 1934), which established fundamental principles for studying electrical activity in nerve fibers and cortical tissue. Adrian’s systematic investigations of nerve fiber activity and cortical electrical spread provided the methodological foundation for subsequent ephaptic coupling studies.

Building on these foundations, Katz and Schmitt (1940) provided the first systematic evidence for electrical interactions between adjacent nerve fibers. Their careful experimental work demonstrated that activity in one fiber could influence neighboring fibers through electrical field effects, establishing that neural communication could occur through mechanisms other than direct synaptic contact.

### 2.2 Arvanitaki’s seminal contributions

Angelique Arvanitaki’s (1942) paper “Effects Evoked in an Axon by the Activity of a Contiguous One” represents a foundational milestone in ephaptic coupling research. Arvanitaki conducted her experiments using squid giant axons, a preparation that offered exceptional experimental accessibility and large electrode placements—features that would be impossible with mammalian neurons of that era. Arvanitaki coined the term “ephaptic interactions” and provided detailed experimental evidence demonstrating how activity in one axon could influence a contiguous resting axon through electromagnetic field effects rather than synaptic transmission. Her methodological approach involved careful measurement of electrical signals from nearby axons using extracellular recording techniques, establishing the foundation for how electromagnetic field interactions could be experimentally detected.

Arvanitaki established key terminology that remains in use today, introducing the concepts of “preephaptic” and “postephaptic” axons to describe the source and target of ephaptic influences. Her work demonstrated that action currents could evoke responses with characteristic delays of 2–5 milliseconds, establishing temporal parameters that distinguished ephaptic from synaptic interactions. This early work suggested that ephaptic coupling could represent a significant mechanism for neural communication. However, the experimental limitations of the era were significant: measuring tiny electrical signals in neural tissue required positioning electrodes with extreme precision, signal-to-noise ratios were poor, and distinguishing true field effects from artifacts was challenging. Moreover, mammalian brain tissue presented far greater technical difficulties than squid giant axons—tissue was more fragile, neurons were microscopic, and extracellular recording from closely spaced neuronal populations was nearly impossible with the electrode technology available. These experimental constraints would set the stage for decades of difficulty in replicating and extending Arvanitaki’s findings.

## 3 Mid-century skepticism and marginalization (1950s)

### 3.1 The Lashley challenge

The mid-century period marked a severe setback for ephaptic field theories. Lashley et al. (1951) conducted what appeared to be a definitive experimental test of electrical field theory by placing metallic conductors (gold foil strips and pins) directly in the visual cortex of monkeys. Their experimental design was intended to disrupt any electrical field effects by creating a Faraday cage effect and current flow through highly conductive metal pathways.

Finding no disruption of visual function despite the presence of these metallic conductors, Lashley et al. concluded that “the action of electric currents, as postulated by field theory, is not an important factor in cerebral integration.” This influential study effectively marginalized electromagnetic field theories of brain function for decades, with the broader neuroscience community largely dismissing ephaptic effects as physiologically irrelevant.

### 3.2 Limited supporting evidence

Eccles and Jaeger (1958) provided some evidence for ephaptic effects on ion currents within the synaptic cleft, demonstrating that electrical fields could influence neural communication at the cellular level. However, this work was overshadowed by the broader skepticism following Lashley’s influential findings, and ephaptic mechanisms were generally relegated to the status of curiosities with minimal functional significance.

A crucial turning point came with J.J. Miller’s pioneering work at the University of British Columbia in the mid-1980s, which provided some of the earliest clear demonstrations of ephaptic interactions in cortical circuitry. Richardson et al. (1984) demonstrated that extracellular field potentials could ephaptically discharge CA1 neurons in the hippocampus, suggesting that field effects may play a role in recruitment and synchronization of neuronal activity. Using the *in vitro* hippocampal slice preparation with careful experimental controls, they established that these effects were distinct from synaptic transmission. In a follow-up study, Turner et al. (1984) extended these findings by showing that ephaptic interactions contributed to paired-pulse and frequency potentiation of hippocampal field potentials, connecting field effects to well-studied forms of synaptic plasticity.

Miller’s work was particularly significant because it demonstrated ephaptic effects in mammalian cortical tissue using rigorous methodology, yet these findings remained largely peripheral to mainstream neuroscience throughout the 1980s and 1990s. It was only decades later, with the emergence of research on theta oscillations and traveling waves in hippocampus, that the functional importance Miller had identified became widely recognized. These later studies helped nail down the importance of field effects and provided compelling evidence for the ability of electromagnetic fields to carry and process information and feed this back to neurons, validating Miller’s earlier insights.

## 4 Modern renaissance (2000s–2010s): technological advances enable new discoveries

The field experienced renewed interest beginning in the 2000s, catalyzed by technological breakthroughs that enabled new classes of experiments impossible with prior tools. Multi-electrode arrays and high-density recording systems allowed simultaneous measurement of local field potentials and single-unit spiking across distributed neural populations, enabling researchers to characterize spatial and temporal relationships between field dynamics and neural activity with unprecedented resolution. Computational modeling capabilities had advanced sufficiently to simulate neural tissue at scales bridging single cells and networks, permitting detailed examination of how field effects could emerge from cellular properties and propagate through tissue. Foundational work by Jefferys (1995) and colleagues throughout the 1980s–2000s had established that nonsynaptic modulation of neuronal activity through electric currents and extracellular ions was a significant phenomenon, providing critical theoretical and empirical support for renewed interest in ephaptic mechanisms. These technological and computational advances enabled the elegant experiments by Frohlich and colleagues, who demonstrated that exogenous electric fields as weak as 0.5 V/m could modulate neocortical network activity in brain slices, and that endogenous fields could mediate self-regenerating neural waves. Such experiments would have been technically impossible in prior decades: earlier single-electrode recordings lacked the spatial resolution to characterize field-dependent network phenomena, and the computational frameworks necessary to interpret such data did not exist.

Anastassiou and colleagues provided crucial evidence that local field potentials could coordinate individual neuron spiking through ephaptic mechanisms, establishing that endogenous electrical fields were not merely epiphenomena but could actively influence neural activity. This work began to establish the physiological relevance of ephaptic interactions in functioning neural networks.

Hales (2014) provided new theoretical grounding for field-primary consciousness theories through his foundational work on the brain’s endogenous electromagnetic fields. He introduced the concept of “electromagnetic correlates of consciousness” (EMCC), proposing that electromagnetic fields operating across multiple spatiotemporal scales constitute the primary substrate of consciousness rather than serving merely as epiphenomena of neural firing (Hales, 2021). Hales emphasized that the brain is fundamentally comprised of nested electromagnetic fields—from nanometer-scale subcellular phenomena to centimeter-scale whole-brain dynamics—each contributing causally to consciousness through their coordinated dynamics. This framework reoriented consciousness research toward the physics of electromagnetic fields themselves, arguing that understanding how the brain generates consciousness requires moving beyond spike-based neural codes to recognize that electromagnetic field dynamics at multiple scales represent the primary computational mechanism. Hales’ conceptual contribution established the theoretical foundation upon which contemporary field-primary theories have been developed (Author Hunt also credits a longstanding collaboration and dialogue with Hales for a significant part of his acquired understanding of EM fields in relation to cognition and consciousness).

In parallel with foundational ephaptic coupling research, the brain stimulation literature provided independent evidence that weak electric fields influence neural activity. Transcranial direct current stimulation (tDCS) studies demonstrated that fields as weak as 0.4–0.8 V/m could modulate behavior and cognition, with effects showing frequency-dependent and state-dependent characteristics consistent with ephaptic mechanisms rather than simple depolarization. Transcranial magnetic stimulation (TMS) and deep brain stimulation (DBS) findings similarly converged on the conclusion that distributed field effects, not merely local circuit activation, explained the behavioral and cognitive outcomes. These clinical and neurotechnological approaches, developed independently from the basic ephaptic coupling literature, validated the functional significance of electric field effects across scales from local circuits to whole-brain systems. This convergence from multiple methodological traditions substantially strengthened the case that ephaptic coupling constitutes a fundamental mechanism of neural computation.

## 5 Breakthrough period (2019–2020): definitive evidence for ephaptic transmission

Chiang et al. (2019) provided the definitive experimental demonstration that catalyzed widespread field acceptance. Their elegant experiments demonstrated that slow oscillatory waves could propagate across surgically severed tissue gaps of up to 400 microns, transmission that would be impossible through synapses (which require continuous membrane contact) or gap junctions (which require direct cytoplasmic continuity). This work provided the first unambiguous proof that ephaptic coupling alone—without any structural connections between tissue regions—could support meaningful, bidirectional information transmission. Complementing this experimental work, Shiffman and Lewis (2019) developed ELFENN, a computational platform for modeling ephaptic coupling in spiking neuron models, providing tools for integrating ephaptic effects into neural simulations. Together, these works demonstrated that ephaptic coupling could be both measured experimentally and modeled computationally, establishing it as a quantifiable neural phenomenon.

Ruffini et al. (2020) advanced the field through sophisticated finite element modeling studies of mesoscopic ephaptic coupling in the human brain. Using realistic head models from 401 subjects, they demonstrated that electric fields generated by transcranial electrical current stimulation are of the same magnitude as endogenous fields, and developed novel methods to estimate ephaptic interaction strength in individual brains. They calculated that ephaptic effects could travel up to 5,000 times faster than synaptic effects.

Herweg et al. (2020) provided important context for understanding ephaptic influences on memory-related theta oscillations, demonstrating how electromagnetic field interactions might contribute to memory formation and retrieval processes.

## 6 Contemporary renaissance and historical rebuttal (2022 onwards)

MacIver (2022) rebuttal identified a critical flaw in Lashley et al.’s (1951) experimental logic. His key insight reveals that ephaptic coupling operates through quasistatic (near-field) electromagnetic fields—not radiative (far-field) effects. Quasistatic EM fields propagate at light speed through neural tissue and permeate the brain’s dielectric medium throughout. They are not directional in the sense of “inward” or “outward,” but rather form field patterns determined by charge and current distributions in neuronal membranes. This distinction is critical: radiative EM requires accounting for propagation delays over large distances, while quasistatic EM operates effectively instantaneously throughout the medium at each point. This explains why Lashley’s surface-level disruption failed—the operative quasistatic fields permeate the entire tissue.

Cunha et al. (2022) provided detailed computational validation of ephaptic mechanisms using the Quadratic Integrate-and-Fire with Ephaptic coupling (QIF-E) model. Their work demonstrated that ephaptic entrainment could be accurately modeled and predicted, providing a quantitative framework for understanding ephaptic interactions.

An important clarification: the electromagnetic field phenomena underlying both action potential propagation and ephaptic coupling arise from a single unified EM field system generated by neuronal activity. The distinction between these mechanisms is not one of different physical sources, but of different propagation directions and spatial scales. Action potential coupling represents longitudinal electromagnetic coupling along the neuronal membrane, while ephaptic coupling represents transverse electromagnetic coupling through surrounding tissue. Both arise simultaneously from the same charge distributions and transmembrane currents in neuronal membranes. Both represent quasistatic electromagnetic field phenomena. Viewing them as a unified field system rather than separate mechanisms provides more physically accurate understanding of neural dynamics.

### 6.1 Consciousness and field theories

Hunt and Jones (2023) conducted a comprehensive examination of evidence for ephaptic field effects and consciousness theories in their study “Fields or firings? Comparing the spike code and the electromagnetic field hypothesis.” This work is significant for consciousness research because it systematically argues that oscillating electromagnetic fields may represent the primary seat of consciousness, operating as a gestalt with synaptic firing and other neuroanatomical features to produce the complexity of conscious experience. Hunt and Jones propose that rather than searching solely for neural correlates based on spike codes, consciousness research should focus on electromagnetic correlates of consciousness (EMCC)—the specific oscillatory field patterns that generate consciousness. For human cortical function, this perspective implies that the unified electromagnetic field system we discussed earlier, generated across multiple scales and arising from both action potential and ephaptic coupling phenomena, may be the actual substrate of our conscious experience, built on top of the neuroanatomical backbone of the brain and body. This reframing places ephaptic coupling at the center of understanding human consciousness, suggesting that the transverse electromagnetic interactions through cortical tissue are not merely supporting players but fundamental to the emergence of conscious awareness.

This paper and MacIver’s were part of a Frontiers in Human Neuroscience research topic on “Electromagnetic Field Theories of Consciousness: Opportunities and Obstacles,” with a downloadable pdf of the topic, plus a summary editorial (Hunt et al., 2024).

### 6.2 Memory network formation

Pinotsis and Miller (2023) provided empirical evidence for ephaptic coupling between cortical regions *in vivo*, demonstrating that ephaptic coupling plays a causal role in local neuronal activity and memory network formation. This work is critically important for the ephaptic communication hypothesis because it shows that electromagnetic field interactions do not just modulate activity incidentally—they actively structure how memory networks form and organize. The authors established that ephaptic effects could be detected and quantified in functioning brain circuits during memory tasks, providing direct evidence that the field-level phenomena we have discussed throughout this review are not just laboratory curiosities but functional components of cognitive processes. Pinotsis et al. (2023) further demonstrated how electromagnetic fields sculpt neural activity and influence memory formation, showing that ephaptic coupling contributes directly to the brain’s computational infrastructure. Together, these works establish that memory network organization—one of the brain’s most fundamental cognitive operations—depends on quasistatic electromagnetic field interactions operating transversely through cortical tissue (Logothetis et al., 2007).

Pinotsis et al. (2023) further demonstrated how electric fields sculpt neural activity and influence memory formation, showing that ephaptic coupling contributes to the brain’s computational infrastructure.

### 6.3 Scale-dependent mechanisms: local and large-scale integration

**Figure 1:**
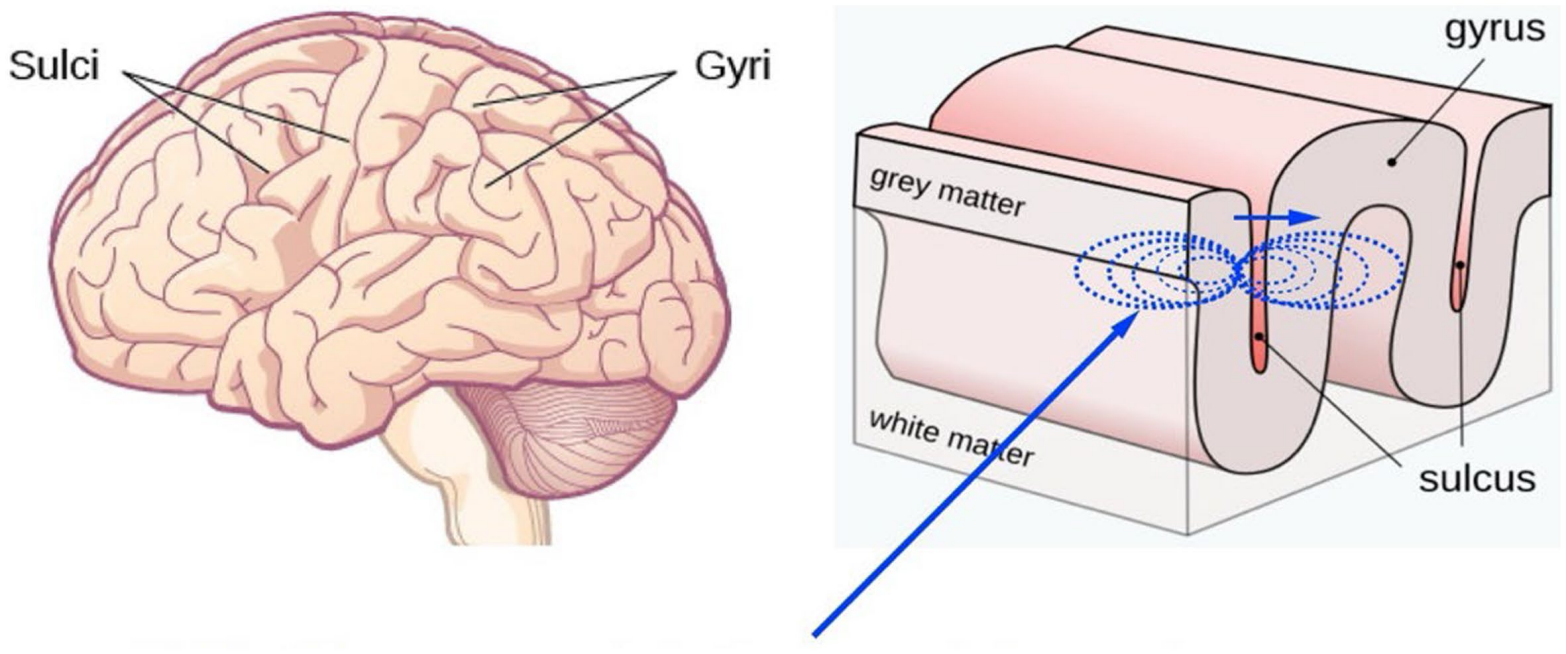
Ephaptic coupling mechanism across cortical sulci. Electromagnetic fields generated by neuronal discharge in one gyrus (shown in blue dotted field lines) can influence the discharge probability of neurons on the opposite side of a sulcus, enabling communication between spatially separated neural populations without direct synaptic connections.

A critical distinction emerging from contemporary research concerns the scale at which ephaptic coupling operates and its functional consequences. Local ephaptic coupling—electromagnetic field interactions spanning 10s to 100s of microns between nearby neural populations (Figure 1)—operates as a rapid integrative mechanism within neural circuits, enabling the coordinated discharge of neighboring neurons without synaptic transmission. This local scale is exemplified by cardiac ephaptic coupling (Gourdie, 2019), where gap junction-independent electrical coupling between adjacent myocardial cells coordinates contraction within confined spatial regions.

**Figure 2:**
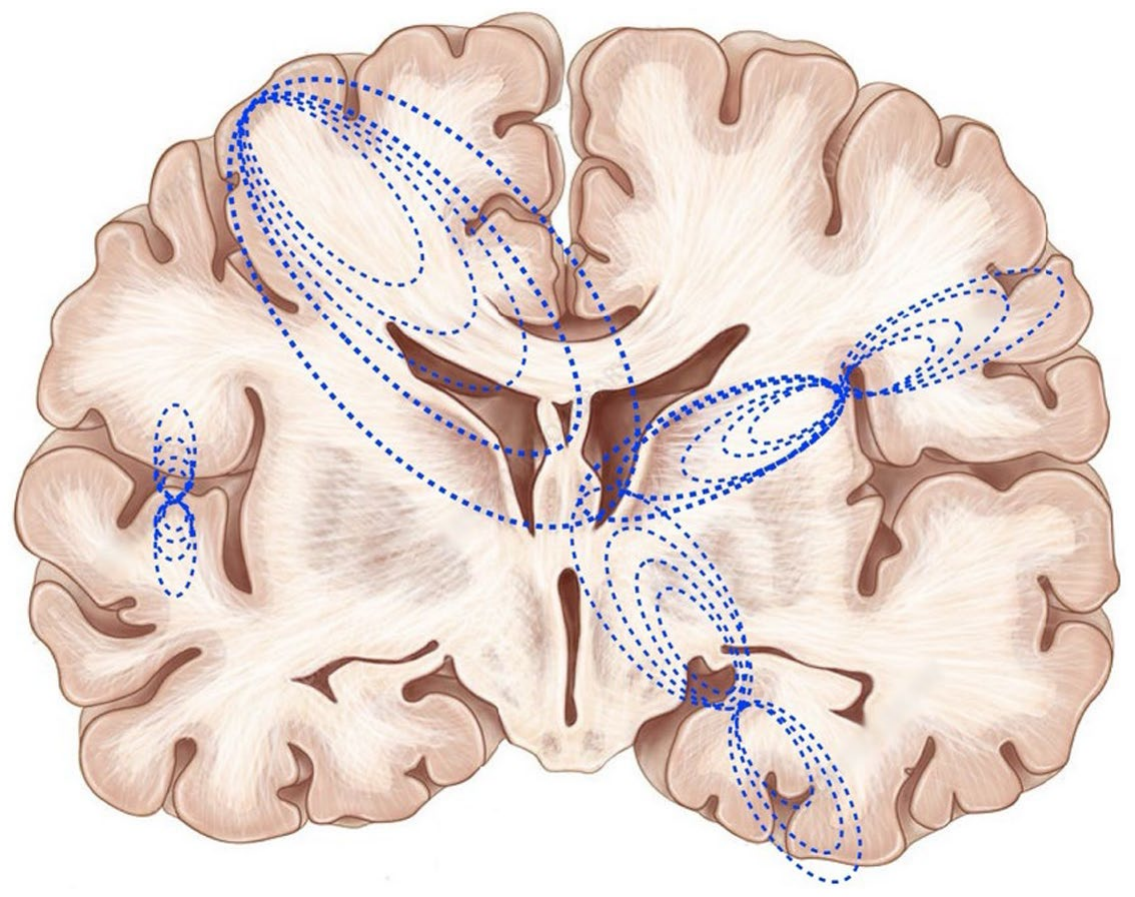
Large-scale ephaptic coupling across brain regions. Electromagnetic field lines (blue dotted curves) illustrate how neuronal activity can generate fields that span across multiple cortical regions and through subcortical structures, contributing to the integration and binding of distributed neural activity to produce a unified conscious experience. This is an oversimplified schematic; in a functioning brain there would be 1000s of these current loops that simultaneously generate a 3D unified EMF, that changes constantly. We postulate that this field is the ‘mind’, generated by the brain and, in turn, able to ephaptically control the discharge of neurons throughout the brain.

In contrast, large-scale ephaptic coupling (Figure 2) operates across millimeters to centimeters, spanning multiple cortical regions and connecting cortical areas to thalamic and subcortical structures. At this scale, electromagnetic field interactions contribute to the integration and binding of distributed neural activity across the entire brain. This distinction is functionally significant: local ephapsis enables rapid local computation and synchronization, while large-scale ephapsis provides the unified field architecture proposed as the neural substrate of consciousness. The seamless transition between these scales—from local circuits generating currents that create large-scale fields, which in turn modulate the local circuits that generated them—creates a hierarchy of electromagnetic field effects that may be essential for the unified conscious experience described by binding problem theories.

### 6.4 Physical mechanisms and scale

Schlötter et al. (2023) provided a detailed analysis of ephaptic coupling mechanisms in their study of action potential annihilation and electrical coupling between neurons. This work is significant for understanding scale-dependent ephaptic effects because it demonstrates that ephaptic coupling effects can span 100s of microns and are ubiquitous across meaningful length and time scales. The action potential annihilation phenomenon—where action potentials can suppress each other through electromagnetic interactions—reveals the multi-scale nature of ephaptic coupling: local electrical events at the scale of a single neuron’s membrane can propagate and interact at regional scales through the tissue’s electromagnetic field. This demonstrates that the distinction we made earlier between longitudinal (action potential) and transverse (ephaptic) electromagnetic coupling operates seamlessly across scales, with local longitudinal events generating fields that interact transversely with distant neuronal populations. Understanding this multi-scale integration is essential for comprehending how the unified electromagnetic field system generates coherent activity across the brain (Holt and Koch, 1999).

### 6.5 Cell-type specific effects

Lee et al. (2024) revealed that neurons in rodent and human cortex exhibit strong, cell-class-dependent entrainment that depends on stimulation frequency. Their work demonstrated that excitatory pyramidal neurons entrain to both slow and fast electric fields, while inhibitory classes like PV and SST predominantly phase-lock to fast fields, providing evidence for selective ephaptic effects on different neuronal populations.

### 6.6 Network complexity and brain function

Cunha et al. (2024) demonstrated that ephaptic coupling increases brain network complexity by 7–13% in their computational study. They showed that “ephapticity is not merely insignificant background noise caused by synaptic activities” but rather “exerts a significant influence on neuronal communication and the brain’s energetic cost.”

Kluger et al. (2024) explored how ephaptic interactions contribute to complex spatiotemporal brain–body trajectories, proposing that interoceptive rhythms and brain dynamics exist within low-dimensional manifolds shaped by electromagnetic field interactions.

### 6.7 Field consolidation

The 2024 Frontiers ebook “Electromagnetic field theories of consciousness: Opportunities and obstacles” (Hunt et al., 2024) brought together leading researchers examining how ephaptic fields may underlie conscious experience, representing a major consolidation of the field’s progress toward understanding consciousness through electromagnetic field theories. This consolidation is significant because it marks the recognition that decades of fragmented research on ephaptic coupling, electromagnetic fields, and consciousness can now be integrated into a coherent theoretical framework. The collection demonstrates that researchers from diverse backgrounds—computational neuroscientists, experimental neurophysiologists, consciousness theorists, and physicists—converge on the understanding that quasistatic electromagnetic fields operating at multiple scales are fundamental to neural computation and consciousness. This convergence suggests we are at an inflection point where field-based approaches transition from marginal to mainstream in neuroscience and consciousness studies.

### 6.8 Supporting research

Throughout this timeline, additional research has provided converging evidence for ephaptic effects across multiple biological systems. Gourdie (2019) demonstrated ephaptic mechanisms in cardiac tissue, showing that gap junction-independent electrical coupling contributes to heart function. Schmidt et al. (2021) showed that ephaptic coupling in white matter fiber bundles can explain sensory stimulus latency effects that cannot be accounted for by other mechanisms.

## 7 Current understanding and future directions

Research spanning six decades has established that weak electric fields (0.1–5 V/m) can produce measurable physiological effects in neural tissue. The field has experienced a remarkable transformation from early promise through decades of marginalization to current recognition that ephaptic interactions provide fast and direct communication between neurons, enabling new mechanisms for communication and computation that complement traditional synaptic transmission.

MacIver (2022) rebuttal has been crucial in explaining why the mid-century dismissal was premature and misinterpreted. This historical correction has facilitated rapid contemporary advances toward practical applications in brain stimulation protocols and fundamental questions about consciousness itself.

Current evidence suggests that ephaptic coupling contributes to multiple aspects of brain function, including memory formation, network complexity, sensory processing, and potentially consciousness. The field is moving toward integration of ephaptic mechanisms into comprehensive theories of brain function and toward practical applications in neurotechnology and therapeutic interventions.

## 8 Conclusion

The history of ephaptic field research demonstrates how scientific paradigms can undergo dramatic shifts as methodological advances enable more sophisticated investigation of complex phenomena. The arc from early enthusiasm through decades of dismissal to contemporary renaissance illustrates the importance of continued scientific inquiry and the willingness to revisit previously dismissed theories when new evidence emerges.

Contemporary ephaptic coupling research has established that electromagnetic field interactions represent a fundamental mechanism of neural communication that operates alongside, but independently from, chemical synaptic, as well as electrical (gap junction) transmission. The recognition that ephaptic effects can influence memory formation, brain network complexity, and potentially consciousness itself suggests that electromagnetic field theories will play an increasingly important role in understanding brain function and developing new therapeutic approaches.

Future research directions include further elucidation of ephaptic mechanisms across different brain regions and cell types, the co-evolution of differential myelination with ephaptic field effects, development of therapeutic applications based on electromagnetic field manipulation, and continued integration of ephaptic coupling into comprehensive theories of consciousness and cognition. An emerging frontier involves applying EM field principles to artificial intelligence and neuromimetic computing, particularly in developing neuromorphic chips that replicate neural electromagnetic field phenomena. The historical trajectory of this field suggests that understanding the brain and consciousness requires grasping the fundamental role of quasistatic electromagnetic fields across all spatial and temporal scales.
